# Cancer-associated Fibroblast-derived IL-6 Promotes Head and Neck Cancer Progression via the Osteopontin-NF-kappa B Signaling Pathway: Erratum

**DOI:** 10.7150/thno.69175

**Published:** 2022-01-21

**Authors:** Xing Qin, Ming Yan, Xu Wang, Qin Xu, Xiaoning Wang, Xueqin Zhu, Jianbo Shi, Zhihui Li, Jianjun Zhang, Wantao Chen

**Affiliations:** 1 Department of Oral and Maxillofacial-Head & Neck Oncology, Ninth People's Hospital, Shanghai Jiao Tong University School of Medicine, Shanghai, 200011, PR China;; 2 Shanghai Key Laboratory of Stomatology & Shanghai Research Institute of Stomatology; National Clinical Research Center of Stomatology, Shanghai, 200011, PR China.

The authors regret that the original version of our paper unfortunately contained some incorrect representative images. The migration image of “CMV-OPN” group in Figure 3G, and the invasion images of “CAL-27-rhIL-6+OPN Ab” group and “SCC-25-rhIL-6+PDTC” group in Figure 7D have been misused during image processing. The authors have repeated the experiments in Figure 3G and 7D, and obtained the consistent results as that in the article. The corrected version of the Figure 3G, 7D and 7E (revised cell count results for Figure 7D) is shown below. The authors confirm that the corrections made in this erratum do not affect the original conclusions. The authors apologize for any inconvenience or misunderstanding that the errors may have caused.

## Figures and Tables

**Figure 1:**
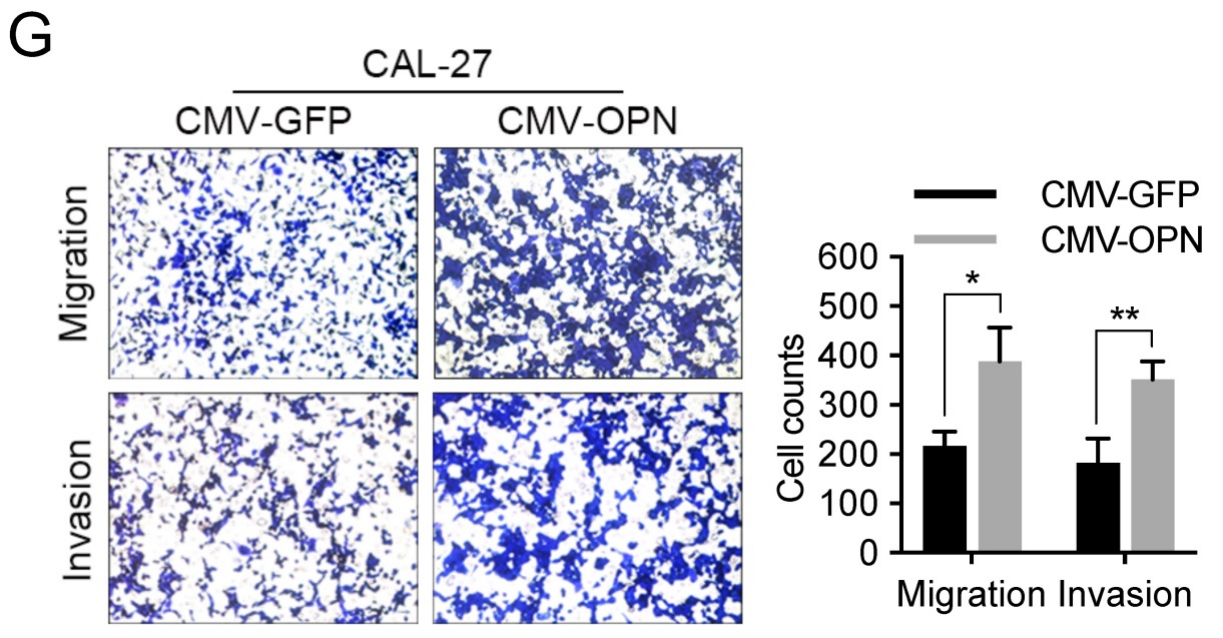
Corrected image for original Figure 3G.

**Figure 2:**
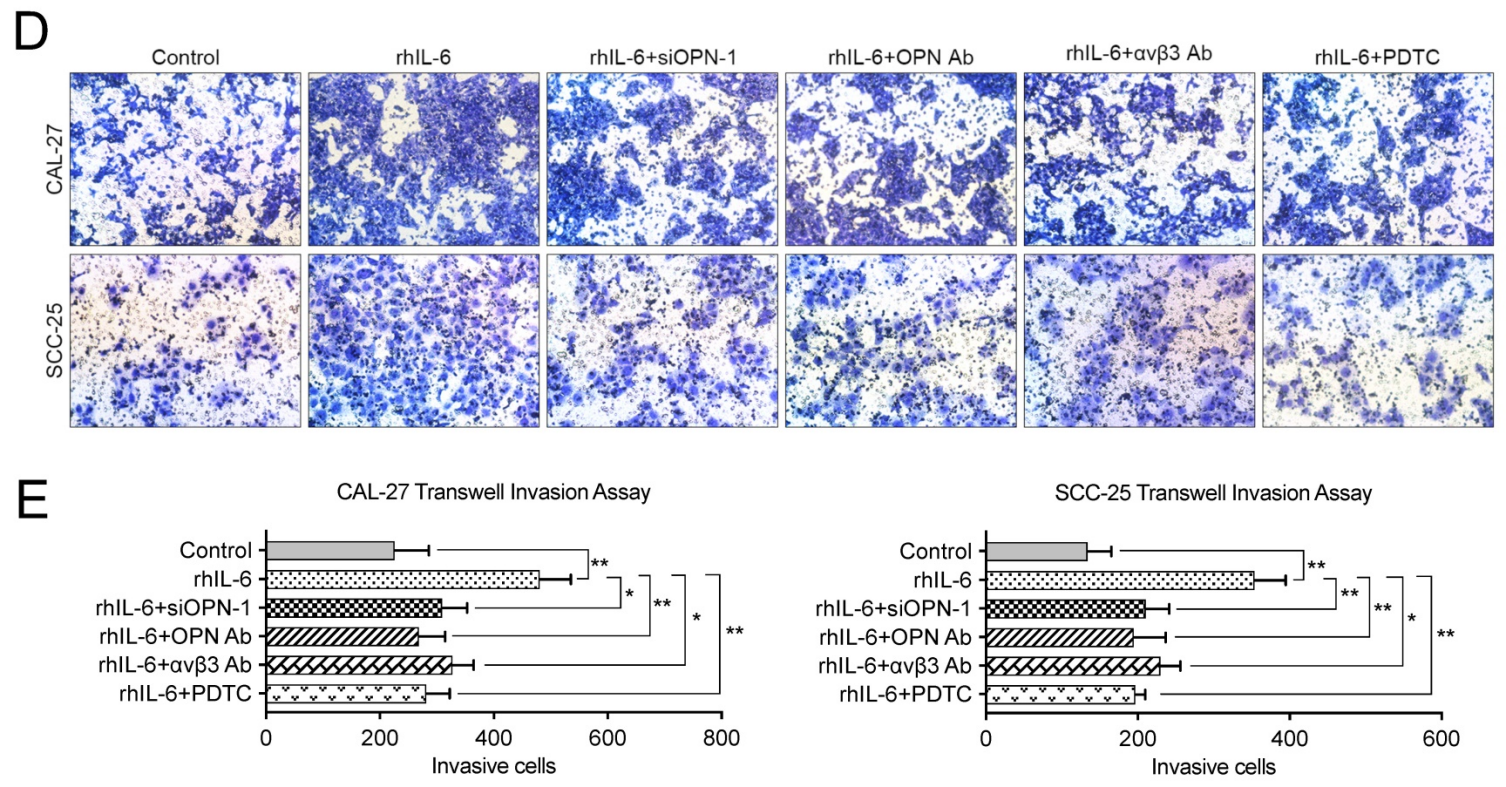
Corrected images for original Figure 7D and E.

